# The Desmosome-Keratin Scaffold Integrates ErbB Family and Mechanical Signaling to Polarize Epidermal Structure and Function

**DOI:** 10.3389/fcell.2022.903696

**Published:** 2022-05-24

**Authors:** Kathleen J. Green, Carien M. Niessen, Matthias Rübsam, Bethany E. Perez White, Joshua A. Broussard

**Affiliations:** ^1^ Department of Pathology, Northwestern University Feinberg School of Medicine, Chicago, IL, United States; ^2^ Department of Dermatology, Northwestern University Feinberg School of Medicine, Chicago, IL, United States; ^3^ Robert H. Lurie Comprehensive Cancer Center of Northwestern University, Chicago, IL, United States; ^4^ Department Cell Biology of the Skin, University Hospital of Cologne, University of Cologne, Cologne, Germany; ^5^ Cologne Excellence Cluster on Stress Responses in Aging-associated Diseases (CECAD), University Hospital of Cologne, University of Cologne, Cologne, Germany; ^6^ Center for Molecular Medicine (CMMC), University Hospital of Cologne, University of Cologne, Cologne, Germany

**Keywords:** epidermal polarity, desmoglein, intermediate filament, EGFR signaling, actin cytoskeleton

## Abstract

While classic cadherin-actin connections in adherens junctions (AJs) have ancient origins, intermediate filament (IF) linkages with desmosomal cadherins arose in vertebrate organisms. In this mini-review, we discuss how overlaying the IF-desmosome network onto the existing cadherin-actin network provided new opportunities to coordinate tissue mechanics with the positioning and function of chemical signaling mediators in the ErbB family of receptor tyrosine kinases. We focus in particular on the complex multi-layered outer covering of the skin, the epidermis, which serves essential barrier and stress sensing/responding functions in terrestrial vertebrates. We will review emerging data showing that desmosome-IF connections, AJ-actin interactions, ErbB family members, and membrane tension are all polarized across the multiple layers of the regenerating epidermis. Importantly, their integration generates differentiation-specific roles in each layer of the epidermis that dictate the form and function of the tissue. In the basal layer, the onset of the differentiation-specific desmosomal cadherin desmoglein 1 (Dsg1) dials down EGFR signaling while working with classic cadherins to remodel cortical actin cytoskeleton and decrease membrane tension to promote cell delamination. In the upper layers, Dsg1 and E-cadherin cooperate to maintain high tension and tune EGFR and ErbB2 activity to create the essential tight junction barrier. Our final outlook discusses the emerging appreciation that the desmosome-IF scaffold not only creates the architecture required for skin’s physical barrier but also creates an immune barrier that keeps inflammation in check.

## Introduction

The appearance of cadherin-based intercellular connections was critical for the acquisition of multicellularity in metazoans (Nichols et al., 2012; Green et al., 2020). Primitive cadherins are thought to have been important for interactions between pre-metazoans and the external environment. Later, epithelial cadherins began to couple cells with each other and the cortical actin cytoskeleton through adherens junctions (AJs). During this time, there was an expansion of receptor tyrosine kinases (RTKs), signaling molecules with broad functions in regulating proliferation, differentiation, and tissue dynamics. Coupling cadherins with RTKs may have allowed metazoans to engage in signal transduction beyond their environment, to neighboring cells (Nichols et al., 2012; Suga et al., 2012; Richter and King, 2013; Chiasson-MacKenzie and McClatchey, 2018). In extant vertebrates, AJs and RTKs are thus intimately connected to regulate intrinsic events associated with tissue morphogenesis and epithelial remodeling.

An important step in creating complex tissues was the appearance of desmosomes, which anchor the IF cytoskeleton to sites of strong cell-cell adhesion and are increasingly appreciated as integrators of chemical and mechanical signaling (Najor, 2018; Muller et al., 2021; Hegazy et al., 2022). These connections arose in primitive form in jawless fish, reaching their highest complexity in terrestrial vertebrates (Green et al., 2020), and are physically and functionally integrated with the actin-cadherin network in vertebrates (Rubsam et al., 2018; Broussard et al., 2021; Prechova et al., 2022). In this mini-review we discuss how overlaying this IF-desmosome network onto the ancient cadherin-actin network exerted further control over RTKs in the ErbB family. Focusing on the epidermal covering of the skin as a model, we discuss how desmosome-IF connections and ErbB family members cooperate to create a gradient of signaling and mechanics critical for the formation of this multi-layered regenerating tissue.

## The Desmosome-IF Network in Epithelia

Desmosomes, along with tight junctions (TJs) and AJs, are one of three cytoskeletal-associated intercellular junctions in simple and stratified epithelial tissues of vertebrates. While TJs and AJs are ancient in origin and associate with the actin cytoskeleton, desmosomes appeared in vertebrates and anchor IF to the plasma membrane. In simple epithelia TJs, AJs, and desmosomes are distributed in polarized fashion along the apical to basal region of the lateral membrane, whereas in the multilayered epidermis these junctions are distributed across multiple layers from superficial (apical) to deep (basal layers) (Niessen, 2007; Niessen and Gottardi, 2008; Rubsam et al., 2018). In both cases, their proper distribution is necessary to generate an effective barrier appropriate for the function of the associated organ (Alizadeh et al., 2021; Muller et al., 2021). Furthermore, these intercellular junctions engage in bi-directional communication with ErbB family members: they contribute to localization and activities of ErbB family members, and in turn, ErbB family members regulate the assembly, integrity, and function of the junctions (Chiasson-MacKenzie and McClatchey, 2018; Muller et al., 2021; Hegazy et al., 2022).

**FIGURE 1:**
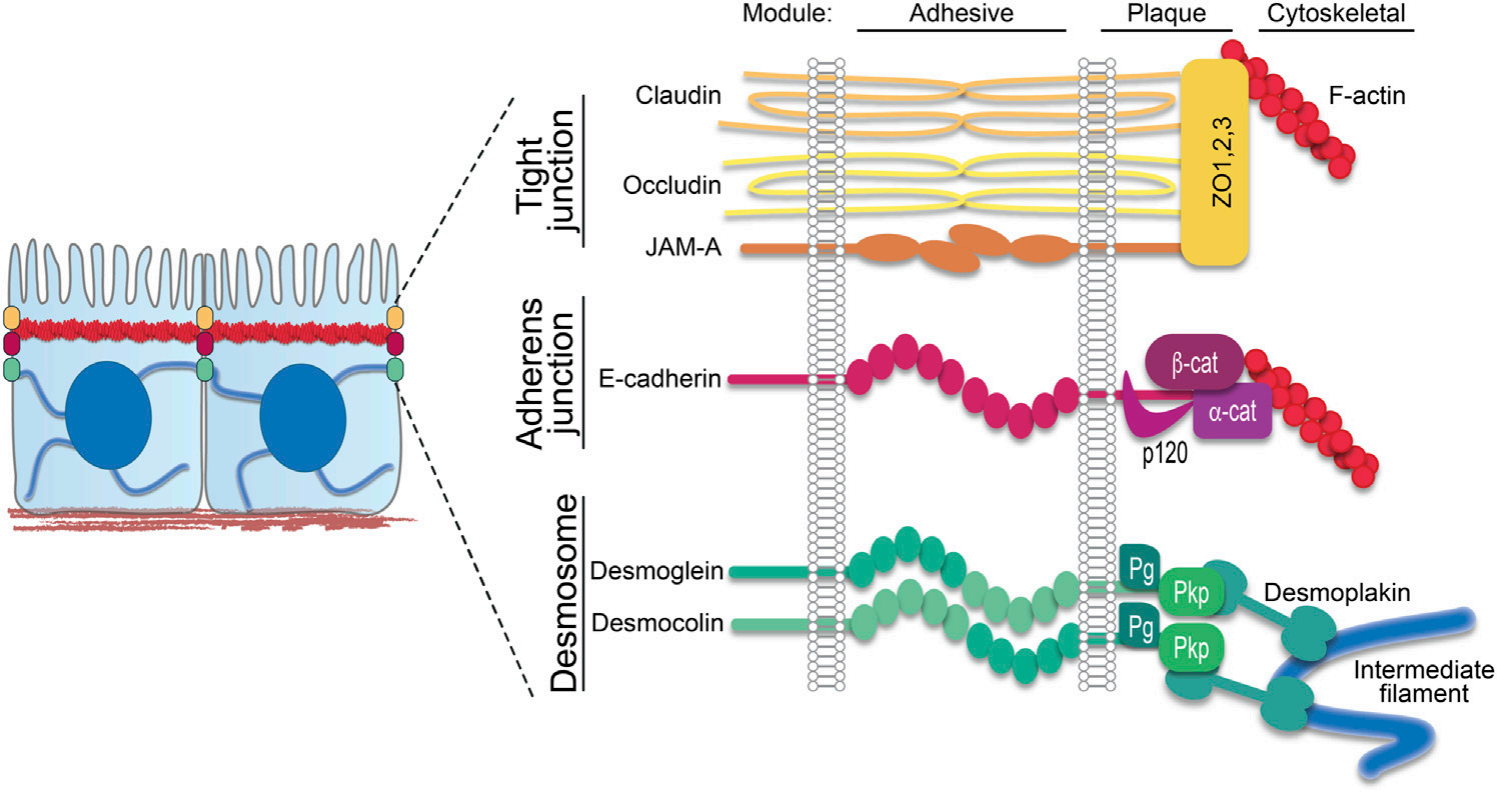
Epithelial junction components. In simple epithelia, the apical junctional complex comprises apical tight junctions followed by adherens junctions, both anchored to the cortical actin ring, and then desmosomes, which anchor the intermediate filament network to the plasma membrane. Schematic shows these three major intercellular junctions associated with cytoskeletal filaments. Transmembrane components span the intercellular space as part of the adhesive core. On the intracellular side, these transmembrane components interact with plaque proteins that in turn anchor their respective cytoskeletal filaments. ZO, zonula occludens; *β*-cat, *β*-catenin; *α*-cat, *α*-catenin; Pg, plakoglobin; Pkp, plakophilin.

**FIGURE 2:**
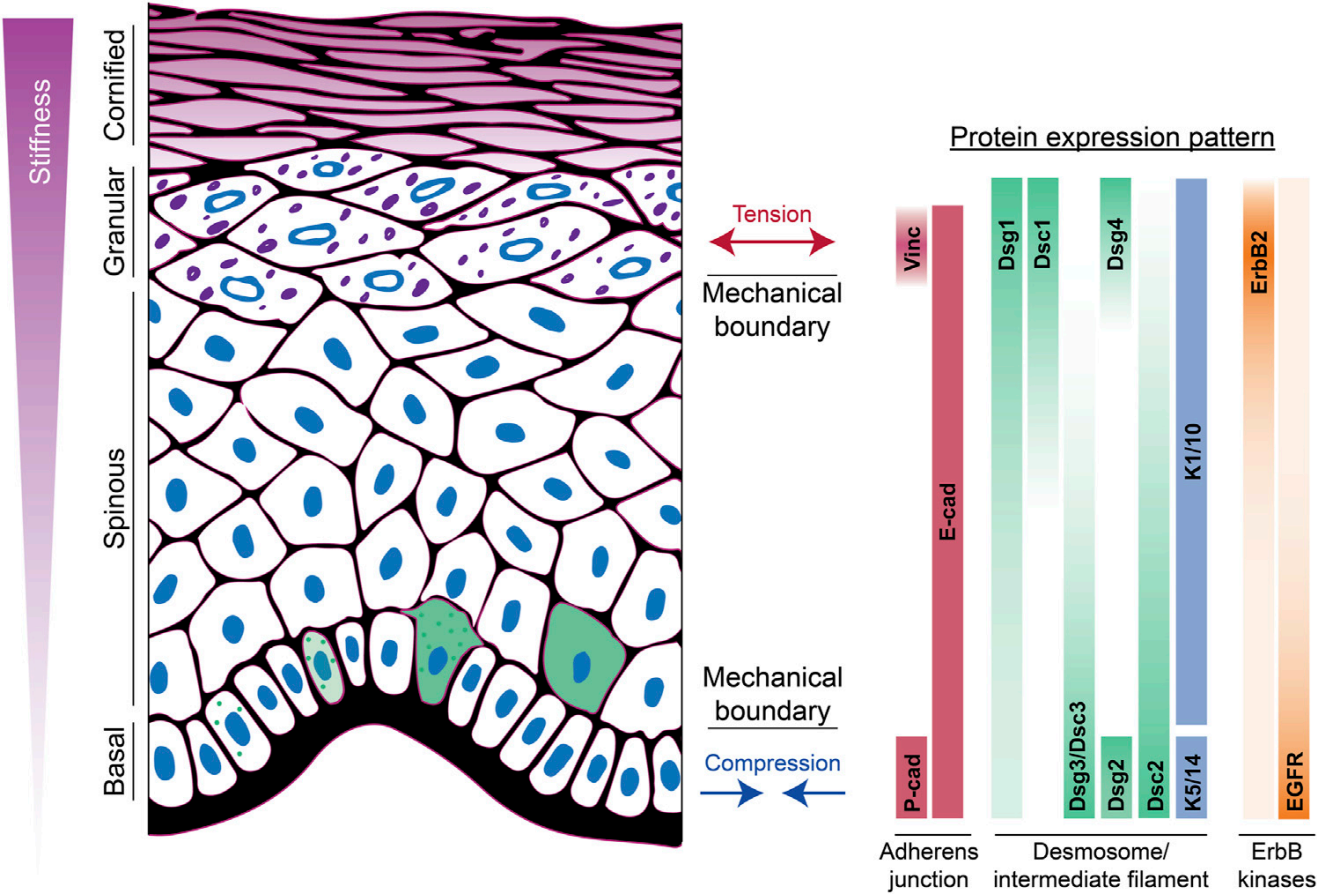
Apicobasolateral polarization of junctions and the cytoskeleton in the epidermis. The spatial distribution of adherens junction, desmosomal, and ErbB tyrosine kinase family proteins across the different layers of the epidermis is shown. This patterned expression is associated with development of mechanical boundaries in both the upper and lower layers. In the basal layer, cells experience compressive forces, while suprabasal layers experience tension. Moreover, there is a stiffness gradient ranging from least stiff in basal cells to most stiff in cornified cells. Green cells indicate onset of Dsg1 expression, induction of differentiation, and movement into the suprabasal layer. Vinc, vinculin; cad, cadherin; Dsc, desmocollin; Dsg, desmoglein; K, keratin.

Desmosomes exhibit a modular organization that parallels actin-associated AJs. Each mediate intercellular adhesion through members of the cadherin family of adhesion molecules and anchor their respective cytoskeletons through a complex of armadillo proteins and specific cytoskeletal adaptors (Figure 1) (Hegazy et al., 2022; Muller et al., 2021; Najor, 2018). Desmosomes stand apart, however, by being built from members of two cadherin subclasses, desmogleins (Dsgs) and desmocollins (Dscs). Simple epithelial desmosomes have one each of these cadherins, Dsg2 and Dsc2, associated with the K8/18 keratin pair. Complex terrestrial tissues like the epidermis have seven different desmosomal cadherin genes expressed in differentiation-dependent, and in some cases reciprocal, gradient patterns (Hegazy et al., 2022) (Figure 2). Why desmosomes have two subclasses of cadherins is not fully understood, but it is notable that the tails of the Dsgs are longer than those of Dscs or of their classical cadherin counterparts, enabling association with several binding partners that mediate multiple specialized functions. Further, *in vitro* experiments suggest that the strongest intercellular binding is exhibited by suprabasal desmosomal cadherins and weakest by basal cadherins (Harrison et al., 2016). Likewise, desmosomal cadherin-associated plakophilins with different adhesive properties and desmosome-associated keratins K5/14 and K1/10 with different mechanical properties are patterned in the epidermis (Muller et al., 2021) (Figure 2). Collectively, these observations suggest that desmosomal protein patterning provides a means of tuning the cellular and supracellular mechanical properties in different layers of complex tissues.

## ErbB Family Members are Patterned in the Epidermis

ErbB1 (EGFR), ErbB2 (Her2), ErbB3, and ErbB4 are a family of RTKs that control cell proliferation, survival, differentiation and cell dynamics through ligand-dependent and -independent signal activation (Nanba et al., 2013a). All ErbB family members are expressed in the epidermis, but their expression and activity patterns differ. This patterning is thought to regulate the balance of proliferation and differentiation, and to control transcription factor networks known to drive the differentiation process, prominently among these being the AP-1 family of transcription factors (Saeki et al., 2012).

Most focus has been on EGFR (ErbB1), which is present throughout the epidermis but exhibits its highest level of activity in the basal layer, where it maintains proliferation and suppresses differentiation through maintenance of Erk signaling (Tran et al., 2012). Total EGFR is also enriched at TJs in the SG2 layer where its activity must be carefully tuned to maintain the TJ barrier (Rubsam et al., 2017). While EGFR deficiency in mice interferes with normal epidermal and hair follicle differentiation (Chen et al., 2016), the fact that these animals are viable raises the possibility of compensation by other family members (Tran et al., 2012). A candidate for such compensation is basally expressed ErbB4; ErbB4 knockout animals exhibited reduced epidermal thickness and proliferation (Hoesl et al., 2018).

ErbB2 has no known ligand and must dimerize with other ErbB family members to exert ligand-dependent mitogenic and pro-tumorigenic activity. While EGFR is concentrated at membranes, ErbB2 is localized in the cytoplasm of basal cells and present at plasma membrane only in superficial cells (Maguire et al., 1989; Stoll et al., 2001; Broussard et al., 2021). Thus, ErbB2 activity may be limited in basal cells by preventing its cell surface localization (Kansra et al., 2002). ErbB3 is present throughout the epidermis with greatest expression in suprabasal and spinous layers (Piepkorn et al., 2003). As ErbB3 has very little catalytic activity on its own, it likely works in concert with suprabasal ErbB2. As will be discussed below, mechanisms must exist in the spinous layers to suppress formation of tight junctions, which are specifically assembled in the second of three stratum granulosum layers (SG2), raising the possibility that ErbB3 may be involved in this suppression.

## Coordinating Cadherin-Based Junctions and ErbB Family Members

RTKs localize to and influence cell-cell contacts to affect local and tissue scale remodeling (Daniel and Reynolds, 1997; Bertocchi et al., 2012; Chiasson-MacKenzie and McClatchey, 2018). Direct or indirect phosphorylation events can weaken protein-protein interactions, stimulate endocytosis and/or promote cadherin turnover and dissolution of junctions (McLachlan and Yap, 2007; McClatchey and Yap, 2012). While most attention has been placed on classic cadherin regulation by RTKs, ErbB signaling also affects desmosomes and their IF attachment through phosphorylation of desmosomal cadherins, armadillo proteins, and plakophilins (Lorch et al., 2004; Yin et al., 2005; Muller et al., 2020; Muller et al., 2021). Inhibiting EGFR can also increase association of the IF-anchoring protein desmoplakin (DP) with desmosomes, and DP phosphotyrosine modifications have been reported (Moritz et al., 2010). ErbB-dependent regulation of junctional proteins can occur directly through the kinase itself or its downstream pathways, which modulate adhesion in multiple contexts including in cancer cells (Lorch et al., 2004; Klessner et al., 2009) and in response to autoimmune antibodies (Spindler et al., 2018). While most observations focus on destabilization of junctions by RTKs, they can also stabilize junctions, suggesting that ErbB signaling can act as a rheostat to dial junctional assembly and function up or down, dependent on the context (Garrod et al., 2008).

Cell-cell contacts also confer critical spatial and mechanical control on RTKs important for their function, both positively and negatively regulating ErbB RTKs (Chiasson-MacKenzie and McClatchey, 2018; Muller et al., 2021). For instance, EGFR recruitment by E-cadherin regulates cell proliferation and differentiation through MAPK activation (Pece and Gutkind, 2000; Fedor-Chaiken et al., 2003). On the other hand, cortical actin-associated Merlin can dampen EGFR activity in contact inhibited cells (Chiasson-MacKenzie et al., 2015), and this relationship is tunable (Kim et al., 2009). Further, loss of E-cadherin, the desmosomal cadherin Dsg1, or keratin IFs elevates EGFR/Erk1/2 signaling (Getsios et al., 2009; Seltmann et al., 2015; Rubsam et al., 2017; Godsel et al., 2022). In the following sections we review how desmosomal cadherin-IF interactions participate in two major aspects of epidermal differentiation: basal cell commitment and maintenance of the TJ barrier.

## Basal Cell Commitment

During vertebrate evolution, the appearance of desmosome-IF connections expanded the mechanisms by which cell-cell contacts exert control on RTKs and supported the transition from simple to complex epithelia. A critical component in this transition is the commitment of basal keratinocytes to differentiate. This process depends on exquisite spatial and temporal coordination of cytoarchitectural and cadherin-based adhesive forces and ErbB family signaling, to create asymmetries in basal cells that determine who stays and who goes into the superficial layer (Rubsam et al., 2018).

Actin remodeling is a key component of this process. In colonies with a mix of stem cells and committed cells, EGF induces a rapid expansion with stem cells on the periphery and committed cells in the middle, each exhibiting a striking difference in actin filament organization. Peripheral stem cells exhibit radial filaments and committed cells exhibit circumferentially organized filaments (Nanba et al., 2013b). In more recent work, differentiating cells in colonies with pattern-delimited boundaries sorted to the center and exhibited changes in mechanics, becoming initially softer than their surrounding neighbors (Miroshnikova et al., 2018). This transition occurs in concert with changes in cadherin expression including the onset of expression of Dsg1, a terrestrial-specific desmosomal cadherin found only in stratified epithelia. Indeed, when Dsg1 is expressed precociously in undifferentiated primary keratinocytes a similar re-organization from radial to peripheral actin occurs. This change occurs in parallel with delivery of an Arp2/3-dependent polymerization complex to Dsg1 and consequent polymerization of actin at desmosomes (Nekrasova et al., 2018). Arp2/3-dependent polymerization is associated with a redistribution of tension on the membrane away from E-cadherin and AJs to drive the process of delamination (Nekrasova et al., 2018).

Dsg1-dependent actin re-organization occurs in concert with changes in classical cadherins, including loss of P-cadherin from the basal layer and increased adhesion in the suprabasal layers as cells delaminate and move into the next superficial layer (Miroshnikova et al., 2018; Rubsam et al., 2018; Broussard et al., 2021). The onset of Dsg1 expression seems to be critical for this process as it is essential for stratification in a 3D human model of epidermal regeneration (Nekrasova et al., 2018; Broussard et al., 2021). Moreover, ectopic expression of Dsg1 is sufficient to promote stratification in simple epithelia that don’t normally express it. Stratification requires attachment of Dsg1-containing desmosomes with IF, as expression of a DP-uncoupling mutant or DP knockdown prevents this process (Broussard et al., 2021). This attachment is also required for the compression of basal cells that occurs during the commitment to stratify and differentiate (Miroshnikova et al., 2018; Broussard et al., 2021).

The onset of Dsg1 expression in committing basal cells coincides with diminished basal Dsg2/3. Next to re-organizing actin, this switch in cadherins dampens EGFR signaling in two ways to promote differentiation. First, by recruiting ErbB2 interacting protein Erbin to the plasma membrane, Dsg1 interferes with Ras-Raf coupling to attenuate Erk signaling associated with the onset of the biochemical program of differentiation (Getsios et al., 2009; Harmon et al., 2013). Second, desmosomes assist the de-neddylating COP9 signalosome (CSN) to shift the balance of neddylation and ubiquitination that promotes EGFR turnover and epidermal differentiation (Najor et al., 2017). While Dsg1 inhibits EGFR, the basal desmosomal cadherins Dsg2/3 have been shown to promote EGFR signaling upon forced expression in suprabasal layers (Brennan et al., 2007). Thus, differential expression of desmosomal cadherins enables the tissue to spatially tune EGFR signaling. Accordingly, the presence of Dsg2/3 but loss of Dsg1 is associated with cancer progression and in some cases poor prognosis (Brown and Wan, 2015; Liu et al., 2021; Muller et al., 2021).

Dampening EGFR activity at the onset of differentiation is likely to be highly integrated with remodeling of cytoskeletal-adhesive complexes to release cells from the basement membrane. Previous work identified a force-activated E-cadherin-dependent signaling cascade that activates integrins and stimulates contraction to induce stiffening, through an EGFR/PI3K-dependent mechanism (Muhamed et al., 2016). While these observations were made in MCF-7 cells, the same principles may apply to basal epidermal cells. In this context, uncommitted basal cells are predicted to exhibit high EGFR activity, greater stiffness than committed cells, and integrin activation. Upon Dsg1 expression, tension on E-cadherin and vinculin recruitment to *α*-catenin is reduced and EGFR signaling is dampened, as cells detach from the basement membrane (Getsios et al., 2009; Nekrasova et al., 2018; Broussard et al., 2021). It seems plausible that the reduced force on E-cadherin may signal to integrins to support this detachment. In addition, as committed cells are “softer” it is expected that alterations in stiffness are orchestrated with reduced tension on E-cadherin and loss of basement membrane attachment (Miroshnikova et al., 2018).

While EGFR internalization and recycling play a critical contributing role in regulating its activity, plasma membrane associated EGFR can be suppressed in contact-inhibited cells through its association with the actin cytoskeleton through the ERM protein family members Merlin and Ezrin (Chiasson-MacKenzie et al., 2015). Here again, onset of Dsg1 expression may play a role as the Dsg1 interacting protein Erbin interacts with Merlin in Schwann cells (Rangwala et al., 2005). Erbin also binds and sequesters the ubiquitin ligase Cbl to inhibit EGFR turnover (Yao et al., 2015). Together, this suggests a model in which Dsg1 recruits Erbin into the actin-rich cortex to dampen EGFR by disrupting protein complexes permissive for EGFR signaling and/or reducing EGFR mobility at the plasma membrane. The latter preserves a non-signaling pool of EGFR poised for activation later in differentiation.

## Creating the Tight Junction Barrier

In simple epithelia, TJs are positioned just apically to the AJ-associated actin belt. Mechanical measurements coupled with genetic interference with AJ versus TJ proteins suggest that AJ and the associated actin belt support high tension in the apical region of the cell, whereas TJ proteins counter or help dissipate these forces (Zihni et al., 2016; Citi, 2019; Rouaud et al., 2020). Likewise, the superficial layers of stratified epithelia are under higher tension than those below (Rubsam et al., 2017; Fiore et al., 2020; Broussard et al., 2021), creating a mechanical boundary that is dependent on the AJ protein E-cadherin and associated with the restricted formation of vinculin-positive AJs and barrier forming TJs in the SG2 layer (Rubsam et al., 2017; Yokouchi and Kubo, 2018). Whether TJs play a similar role in dissipating contractile forces exerted by actin in the epidermis is not known.

While the mechanical boundary between the SG2 layer and the one above precludes the formation of AJs on the apical SG2-SG1 interface, Dsg1-containing desmosomes continue into the SG1 layer that lack AJs. The loss of Dsg1 results in skin peeling due to separation between the SG and cornified layers (Kugelmann et al., 2019; Godsel et al., 2022), highlighting the importance of this distribution for epidermal integrity. Nevertheless, the desmosome-IF network, mediated by superficial Dsg1, appears to work in concert with E-cadherin to restrict TJs and high apical tension to the superficial layers of human reconstructed epidermis (Rubsam et al., 2017; Broussard et al., 2021).

Tuning the relative activities of EGFR and ErbB2 through cadherin-cytoskeleton interactions is critical for restricting TJs to the tension-high SG2 layer of the epidermis. Collectively, data from animal models and reconstructed human epidermis suggest that dampening EGFR/Erk signaling via E-cadherin and maintaining ErbB2 activity via Dsg1 are required for TJ maintenance and function (Rubsam et al., 2017; Broussard et al., 2021). Given that ErbB2 cannot bind ligand without heterodimerizing with another family member, these data raise the possibility that ErbB2 activity is controlled in a ligand-independent manner in superficial epidermis, possibly through phosphorylation by Src-family kinases (Broussard et al., 2021). Another question is how Dsg1 maintains high levels of ErbB2 in the SG2 layer. The fact that Dsg1 binds to Erbin (Harmon et al., 2013), which stabilizes ErbB2 in breast cancer cells (Tao et al., 2014), raises the possibility that Erbin stabilizes ErbB2 in the proximity of Dsg1 in the superficial epidermis. Another open question is how TJ assembly is prevented in the spinous layers. Evidence suggests this could be due at least in part through EGFR-dependent turnover of the TJ protein occludin (Rubsam et al., 2017). In this regard, it is interesting to note that levels of ErbB3 are highest in suprabasal and spinous layers where TJs are not formed, raising the possibility that EGFR-ErbB3 heterodimers play a role in this process. Thus, in addition to there being a mechanical boundary at SG2, there may also be an ErbB activity boundary that contributes to the dynamics of TJ proteins in different layers.

## Outlook: Role of Desmosome-IF Network in Sensing and Responding to Stress

Properly tuned ErbB signaling is critical for normal skin homeostasis. While elevated ErbB signaling is associated with chronic inflammatory disorders, EGFR helps mount innate immune responses and epidermal loss of EGFR or EGFR receptor inhibitors used in cancer treatment elicit adverse inflammatory rashes (Pastore et al., 2008; Lichtenberger et al., 2013; Mascia et al., 2013; Huang et al., 2021). Given the role of cadherin-cytoskeletal networks in controlling ErbB signaling, this raises the question of whether altered signaling resulting from a damaged desmosome-IF network contributes to an immune response.

Indeed, it is increasingly appreciated that interfering with desmosome-IF networks results in inflammation (Bar et al., 2014; Hatzfeld et al., 2017; Scheffschick et al., 2019). Desmosome mutations result in a systemic disorder called SAM syndrome, characterized by severe dermatitis, allergies and metabolic wasting (Samuelov et al., 2013; Polivka et al., 2018; Muller et al., 2021). Likewise, the presence of pro-inflammatory cytokines in the serum of patients with epidermolysis bullosa (EB) suggests that it is not a skin-limited disorder (Annicchiarico et al., 2015; Esposito et al., 2016). While these inflammatory disorders elicit a range of pro-allergic and pro-inflammatory mediators, recent studies uncovered a psoriasis-like IL-17/23 signature in Dsg1-deficient patients and knockout animals (Godsel et al., 2022). Notably, isolated patient keratinocytes express pro-inflammatory cytokines and an inflammatory gene signature was present in Dsg1-deficient mouse embryos prior to any exposure to the environment. These observations indicate an intrinsic role for the desmosome-IF system independent of its physical role in maintaining the barrier.

Mechanisms linking ErbB signaling to inflammation caused by desmosome-IF damage are beginning to emerge. While Dsg1-Erbin complexes promote differentiation through Erk suppression, chronic Dsg1 loss contributes to Erbin displacement, which promotes NFkB-driven inflammatory gene expression (Polivka et al., 2018). Further, keratin loss stimulates itch-inducing TSLP through amphiregulin/EGFR signaling (Scheffschick et al., 2019). Importantly, a cell-based drug discovery assay identified inhibition of cell stress responses that target the EGFR pathway as a new approach to treatment of EB simplex (Tan et al., 2021), and treatment with anti-IL-23/12 inhibitors of Dsg1-deficient humans resulted in remarkable improvement of their disease (Godsel et al., 2022).

In closing, IF-desmosome networks link chemical and mechanical signaling in the epidermis to drive epidermal morphogenesis and barrier function. They also serve as a guardian of the immune barrier, sensing and responding to environmental stress, at least in part through mechanisms that transcend their physical roles in maintaining the epidermal barrier.
